# Experience of menopause across ethnic groups: mapping the evidence through a scoping review

**DOI:** 10.3389/frph.2025.1732836

**Published:** 2025-12-17

**Authors:** Jesús Endara-Mina, Lisseth Coloma-Ramirez, Cristopher-Josue Escudero, Katherine Andrade-Travez, Cristopher-Jordan Osorio, Erika Campaña, Kelly Chicaiza, Magaly Inga, Paulina Ríos-Quituizaca

**Affiliations:** 1 Facultad de Ciencias Jurídicas y Políticas, Universidad Técnica Particular de Loja (UTPL), Loja, Ecuador; 2 ECUAVOLCAN Research Group, Centro de Investigación Para la Salud en América Latina (CISeAL), Facultad de Medicina, Pontificia Universidad Católica del Ecuador (PUCE), Quito, Ecuador; 3 Scientific Association of Medical Students, Universidad Central del Ecuador (UCE), Quito, Ecuador; 4 Facultad de Ciencias Sociales y Jurídicas, Universidad Internacional de Valencia, Valencia, Spain; 5 Facultad de Ciencias Médicas, Carrera de Enfermería, Universidad Central del Ecuador (UCE), Quito, Ecuador; 6 Facultad de Salud y Bienestar, Carrera de Enfermería, Pontificia Universidad Católica del Ecuador (PUCE), Quito, Ecuador; 7 Facultad de Ciencias de la Salud Eugenio Espejo, Carrera de Medicina, Universidad Técnica Equinoccial (UTE), Quito, Ecuador; 8 Facultad de Ciencias Médicas, Carrera de Obstetricia, Universidad Central del Ecuador (UCE), Quito, Ecuador

**Keywords:** menopause, perception, ethnic groups, cultural diversity, women's health

## Abstract

**Background:**

Menopause is a universal biological event whose experience is shaped by cultural and ethnic factors. The available literature reveals a wide range of perspectives across contexts and population groups, including women from diverse ethnic backgrounds. However, differences persist in how symptoms are conceptualized, expressed, and managed according to sociocultural environments. This scoping review aims to map the existing evidence on menopausal experiences among different ethnic groups and to identify recurring thematic patterns.

**Methods:**

The review followed the Joanna Briggs Institute methodology for scoping reviews and was reported in accordance with PRISMA-ScR guidelines. Articles were retrieved from seven databases—Medline/PubMed, Web of Science, and Scopus—using database-specific search strategies. No language or time restrictions were applied. Studies were analyzed descriptively, and quality appraisal was conducted following the interpretive criteria proposed by Dixon-Woods et al.

**Results:**

Out of 446 initial records, 374 remained eligible for title and abstract screening after duplicate removal; 63 full texts were assessed, and 20 studies met the inclusion criteria. Ethnic differences were observed in both the prevalence and interpretation of symptoms: African American and Hispanic women exhibited a greater emotional and vasomotor symptom burden, whereas Asian and Indigenous women tended to frame the menopausal transition as a natural or developmental process.

**Conclusion:**

This scoping review highlights that menopause is not merely a biological phenomenon but a culturally embedded experience shaped by ethnicity, belief systems, and social position. Substantial ethnic differences exist in the perception, reporting, and meaning attributed to menopausal symptoms.

## 1 Introduction

Menopause, a universal physiological transition marking the end of reproductive capacity, has often been approached from a biomedical perspective that emphasizes hormonal decline and symptomatology (1, 2). However, recent studies highlight that the experience of menopause is profoundly influenced by sociocultural context, ethnicity, and belief systems (3–6). Across societies, cultural narratives shape whether menopause is perceived as a natural life stage, a loss of femininity, or a moment of empowerment and wisdom (7).

Despite its global relevance, most menopause research remains concentrated in high-income Western populations, primarily among White or Caucasian women (8). This narrow focus limits understanding of how women from diverse ethnic backgrounds experience menopausal changes, interpret symptoms, and access healthcare. Ethnic identity intersects with other determinants—such as socioeconomic status, education, and migration history—to shape the meanings attributed to menopause and the ways in which symptoms are expressed and managed (5).

Nevertheless, the global evidence base remains fragmented and methodologically heterogeneous. Few reviews have systematically compared ethnic variations in the menopausal experience, and existing studies differ in how they conceptualize ethnicity, measure symptoms, or analyze psychosocial determinants (9). As a result, there is a need for a comprehensive mapping of current evidence to identify gaps, thematic patterns, and research priorities.

Accordingly, this scoping review seeks to synthesize and map the existing literature on women's menopausal experiences across different ethnic groups, emphasizing how cultural, social, and environmental factors influence perceptions, symptom reporting, and quality-of-life outcomes.

## 2 Methods

This review was conducted following the Joanna Briggs Institute's scoping review methodology (10) and reported using the Preferred Reporting Items for Systematic Reviews and Meta-Analyses extension for Scoping Reviews (PRISMA-ScR) (11) (Supplementary Table S1).

### 2.1 Definitions

#### 2.1.1 Ethnicity

Ethnicity is understood as a social group whose members share a sense of collective identity grounded in a common—whether real or perceived—ancestry, and expressed through shared cultural elements such as language, religion, values, practices, or a common history that distinguish them from other groups within a society (12). In the context of this review, ethnic classification followed the self-identification reported in the included studies, grouping participants into broad categories recurrently used in the international literature: African descent, Hispanic/Latina, Asian, Indigenous, Arab, and Caucasian. This approach acknowledges the relational and socially constructed nature of ethnicity, extending beyond biological or racial criteria (13).

#### 2.1.2 Menopause

Menopause is defined as the permanent cessation of menstruation resulting from the loss of ovarian follicular activity, confirmed after twelve consecutive months of amenorrhea with no other pathological cause (14).

#### 2.1.3 Climacteric syndrome

Set of signs and symptoms resulting from the interaction between sociocultural, psychological and endocrine factors occurring in aging women. Its diagnosis is clinical in women with the expected age group for ovarian hypofunction (15).

#### 2.1.4 Perimenopause

Refers to the transition period between the onset of ovarian dysfunction and menopause, extending up to one year after the last menstrual period. As a significant turning point in a woman's life, perimenopause is often associated with vasoconstriction and changes in the urinary and reproductive systems (16).

### 2.2 Search strategy

A reproducible search strategy was implemented using a combination of Medical Subject Heading (MeSH) terms and natural language in titles and abstracts, focusing on *menopause*, *perceptions*, *attitudes*, and *ethnicity* (Table 1). Search terms were combined using Boolean operators to align with the study objectives. References were exported to Rayyan Web and duplicates were manually removed (17). The search strategy aimed to identify studies exploring how women from different ethnic or cultural groups perceive and experience menopause, including sociocultural meanings, attitudes toward symptoms, and coping mechanisms.

**Table 1:** Bibliographic search strategy.

Concept	MeSH terms	Free terms/natural language	Boolean combination
Menopause	“Menopause”[MeSH] OR “Climacteric”[MeSH] OR “Perimenopause”[MeSH]	menopause* OR menopausal transition OR postmenopause* OR climacteric* OR perimenopaus* OR menopausal experience* OR menopausal perception*	(Menopause OR Climacteric OR Perimenopause)
Perceptions/Experiences	“Attitude to Health”[MeSH] OR “Health Knowledge, Attitudes, Practice”[MeSH] OR “Health Behavior”[MeSH]	perception* OR experience* OR belief* OR attitude* OR understanding* OR meaning* OR knowledge* OR emotional experience* OR cultural perception*	(Attitude OR Experience OR Belief OR Perception)
Ethnicity/Culture	“Ethnic Groups”[MeSH] OR “Cultural Characteristics”[MeSH] OR “Minority Groups”[MeSH] OR “Race Factors”[MeSH]	ethnicity OR ethnic group* OR race OR racial difference* OR cultural factor* OR cultural belief* OR indigenous OR Afro-descendant OR Asian OR Latina OR African American OR migrant women	(Ethnic* OR Cultur* OR Race OR Indigenous)
Health context	“Women's Health”[MeSH] OR “Reproductive Health”[MeSH] OR “Quality of Life”[MeSH]	women's health OR midlife women OR middle-aged women OR aging women OR gender differences OR reproductive aging OR quality of life	(Women* OR Health OR Reproductive)

MeSH, Medical Subject Headings.

### 2.3 Information sources

Articles were retrieved from seven databases: Medline/PubMed, Web of Science and Scopus, using specific search equations developed for each database (Supplementary Table S2). No time or language restrictions were applied in order to include all relevant studies addressing ethnic or cultural perceptions of menopause across different historical and social contexts.

### 2.4 Study selection

**Figure 1:**
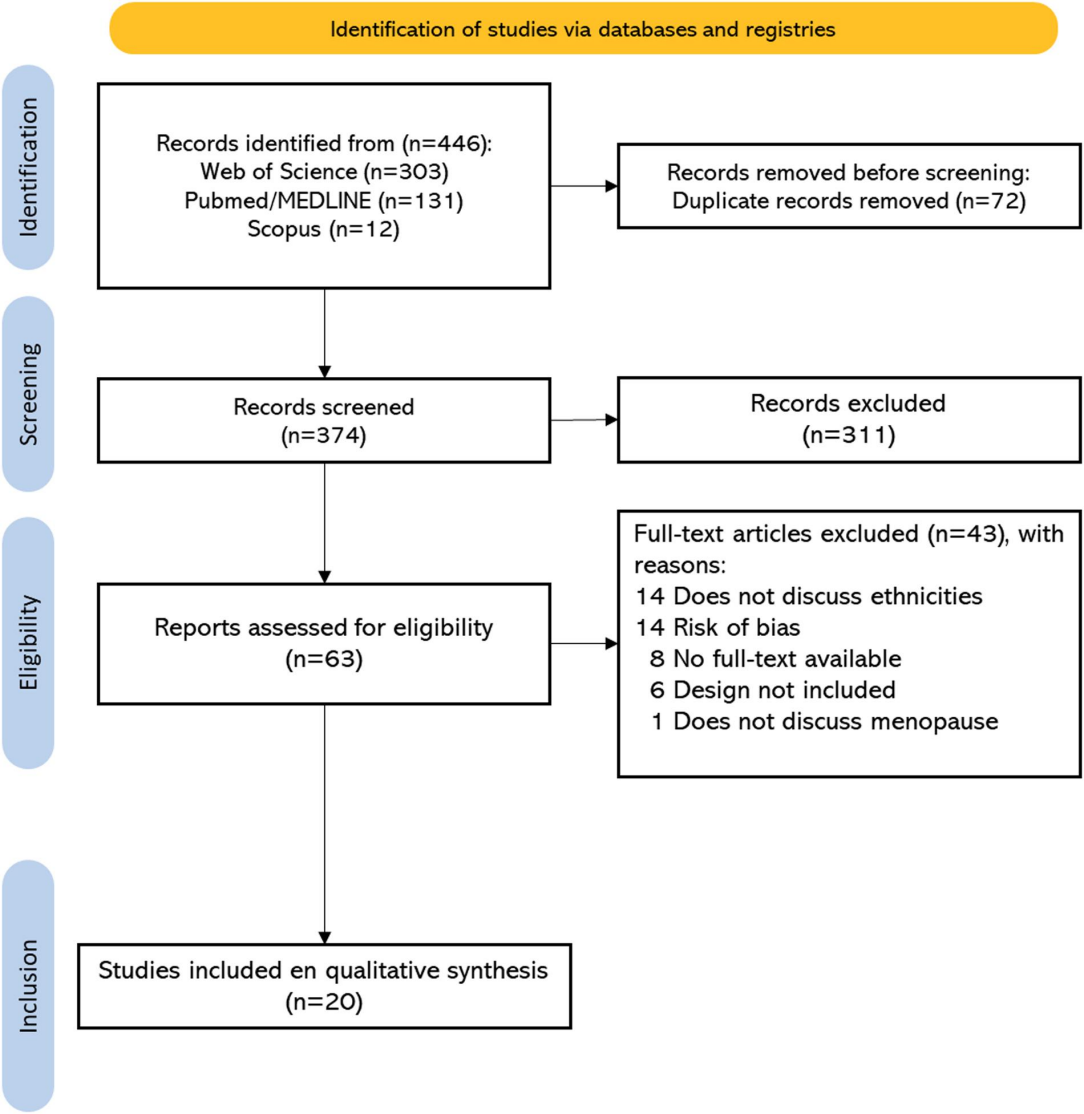
PRISMA flow diagram of the study selection process.

In the first stage, **t**wo reviewers (A-TK and OC-J) independently screened titles and abstracts. Discrepancies were resolved through discussion or by a third reviewer (E-MJ) with expertise in qualitative and cross-cultural health research. In the second stage, full texts were reviewed following the same procedure, and a PRISMA-ScR flowchart (Figure 1) summarizes the selection process. The online Rayyan Web software was used for the screening process (17).

Inclusion criteria encompassed studies addressing perceptions, attitudes, beliefs, or experiences of menopause among women who self-identify with specific ethnic or cultural groups (e.g., Indigenous, Afro-descendant, Asian, or others, according to the national context). Studies using qualitative, quantitative, or mixed methods were included. Exclusion criteria comprised reviews, commentaries, editorials, case reports, conference abstracts, governmental documents, animal studies, and articles without full-text access. Studies focusing solely on biological, hormonal, or clinical aspects of menopause without addressing cultural or perceptual dimensions were excluded.

### 2.5 Data extraction and management

Two reviewers (A-TK and E-MJ) developed a data extraction form refined by the author team. Data were independently extracted by reviewers (C-RL, A-TK, and OC-J), and discrepancies were resolved by a fourth reviewer (E-MJ). Extracted variables included: author(s), publication year, study objective, country, ethnic groups, study design, participant age, analytical approach, and main findings.

### 2.6 Data analysis and synthesis

Studies were analyzed for their methodological approaches, populations, and key findings regarding cultural understandings of menopause. Emergent themes included symbolic meanings of menopause, sociocultural determinants of attitudes, coping strategies, and the intersection of ethnicity and health beliefs. Results are presented in tables summarizing the characteristics and thematic patterns of the included studies.

### 2.7 Quality assessment

The quality of the included studies was assessed using the interpretative synthesis criteria for vulnerable populations proposed by Dixon-Woods, et al. (18) (Supplementary Table S3). Studies failing to meet at least one of the five criteria were excluded. Articles were reviewed by E-MJ and cross-checked by C-RL, with discrepancies resolved through discussion among three reviewers and a third (R-QP) as needed.

## 3 Results

The initial search identified a total of 446 publications. After removing duplicates, 374 studies were eligible for title and abstract screening. Subsequently, 63 full-text studies were reviewed, and 20 studies met the inclusion criteria. The selection process adhered to the previously established eligibility criteria. A summary of the bibliographic search and study selection is provided in Figure 1.

### 3.1 Characteristics of the studies

Table 2 summarizes the general characteristics of the included studies, categorized by ethnic representation, study design, analytical approach, and key findings related to menopausal experiences. Most studies employed quantitative methodologies—primarily cross-sectional designs—while a smaller number adopted longitudinal or comparative/multinational approaches. Only a few incorporated qualitative or mixed-methods analyses focusing on the interpretive and cultural dimensions of menopause.

**Table 2:** General characteristics of the included studies.

Category	Subcategory	Number of studies (n)	Percentage (%)
Ethnic group represented	African American/Black/African	11	–
	Hispanic/Latin American	9	–
	Asian (Chinese, Lebanese, Japanese, Indian, Korean)	12	–
	Indigenous (e.g., Māori, Andean, Native American)	2	–
	Arab/Middle Eastern	3	–
	European/Caucasian/White	13	–
Study design	Cross-sectional	14	70
	Longitudinal/Prospective	3	15
	Comparative/Multinational	3	15
Methodological approach	Quantitative	15	75
	Qualitative	2	10
	Mixed quantitative–qualitative	3	15
Cultural and contextual themes	Menopause as natural stage/life transition	9	45
	Menopause associated with illness or weakness	6	30
	Empowerment/social respect	3	15
	Impact of migration or acculturation	2	10
Geographic distribution	North America	11	–
	Asia	5	–
	Africa	3	–
	Latin America	1	–
	Europe	2	–
	Oceania	1	–

Although 20 studies were included in total, several addressed more than one ethnic group and/or were conducted in multiple countries. Therefore, counts by ethnicity and geographic distribution represent the number of mentions or representations rather than unique studies. Percentages were not calculated for these categories due to regional overlap.

The study populations predominantly included middle-aged women between 40 and 60 years of age, although some extended participation up to 90 years or included perimenopausal groups. Ethnic classification was generally broad, encompassing African American, Hispanic/Latina, Asian (Chinese, Japanese, Indian, Korean), Indigenous (Māori, Andean), Arab, and Caucasian women, often for comparative purposes. However, some studies also explored intra-group differences, such as variations between migrant and native Asian women.

Regarding analytical focus, most studies reported descriptive or ethnicity-stratified analyses, identifying variations in the prevalence, intensity, and impact of symptoms on quality of life. Others applied multivariate regression or factor-analytic methods to assess associations between ethnicity and symptom burden, while a few adopted thematic qualitative approaches to interpret subjective experiences, meanings, and cultural beliefs surrounding menopause.

The most frequently reported outcomes across studies were psychological and vasomotor symptoms, followed by sexual, musculoskeletal, and sleep disturbances. Some studies also examined perceived health deterioration, empowerment, or social respect as cultural expressions of menopause. Taken together, these findings underscore the predominance of biomedical symptomatology in the existing literature, with comparatively fewer investigations addressing the cultural and relational meanings of the menopausal transition.

### 3.2 Critical appraisal

Of the 63 full-text studies assessed for methodological quality and cultural sensitivity, 20 met all inclusion criteria—including the five interpretive criteria proposed by Dixon-Woods et al.—and were included in the final synthesis. The remaining 43 studies failed to meet at least one inclusion criterion; among these, 14 were excluded from the main analysis due to a high risk of bias, specifically for not fulfilling the Dixon-Woods interpretive criteria. The most common reasons for exclusion were the absence of a clearly defined focus on ethnic or cultural variation in the menopausal experience (Criterion 1) and insufficient methodological transparency to support interpretive validity (Criterion 4) (Supplementary Table S3).

### 3.3 Synthesis of findings

The final synthesis included 20 studies published between 1977 and 2022, representing research conducted across six world regions: North America, South America, Europe, Asia, Africa, and Oceania. North America accounted for the largest representation (11 studies), reflecting the predominance of U.S.-based data in the literature. Asian countries such as China, India, and Singapore contributed five studies, while Africa was represented by three studies conducted in Nigeria and Egypt. Europe contributed two studies, and single studies were identified from South America (Bolivia) and Oceania (*N*ew Zealand). Several investigations included participants from multiple countries, underscoring the transnational scope of the evidence base.

Across the 20 studies, more than 20,000 women aged between 35 and 90 years were included, with sample sizes ranging from small qualitative cohorts (*n* = 14) to large multicenter surveys (*n* > 16,000). Most investigations employed cross-sectional designs (70%), followed by longitudinal (15%) and comparative or multinational studies (15%). Collectively, the studies revealed considerable ethnic and cultural variability in menopausal experiences, with frequent comparisons among African American, Hispanic, Asian, Indigenous, Arab, and Caucasian women.

### 3.4 Vasomotor and somatic symptoms

Vasomotor manifestations such as hot flushes, night sweats, and palpitations were the most frequently reported symptoms across ethnic groups, though their prevalence and perceived severity varied considerably. Studies in African American and Hispanic women consistently described a higher frequency and intensity of vasomotor symptoms compared with White or Asian women (19–21). Similarly, Indigenous women from Bolivia identified hot flushes and genital discomfort as their most distressing experiences, often linked to anxiety and fatigue (22).

Somatic symptoms, including joint and muscular pain, fatigue, and physical exhaustion, were also highly prevalent among Arab women in Egypt (23) and among Chinese and migrant Asian women in Germany, where musculoskeletal discomfort was often attributed to both aging and cultural stressors associated with migration (24). In India, menopausal fatigue and joint pain were found to co-occur with psychological symptoms such as irritability and anxiety, forming an interconnected somatic–emotional cluster (25).

### 3.5 Psychological and emotional experiences

Psychological dimensions of menopause—particularly mood changes, irritability, depression, and anxiety—emerged as universal across studies. African American and Hispanic women tended to report more emotional instability than their White counterparts (21, 26). The Study of Women's Health Across the Nation (SWAN) demonstrated that Caucasian women were more likely to report psychosomatic symptoms, while African American women described more vasomotor and stress-related complaints (19, 27).

In Asian populations, fatigue, irritability, and loss of concentration were common, but emotional disturbances were often normalized or considered a temporary imbalance rather than illness (25, 28). Among Arab and African women, feelings of anxiety, sadness, and diminished vitality were associated with the physical discomfort of menopause and social expectations regarding aging and femininity (23, 29). Conversely, some Indigenous and rural groups interpreted emotional calmness post-menopause as a sign of maturity or relief from reproductive obligations (22, 30).

### 3.6 Sexual and relational aspects

Sexual and relational changes emerged as a consistent theme across the included studies, although with substantial variation depending on cultural norms, the symbolic meanings attributed to menopause, and the social positioning of women within each ethnic group. Physical symptoms affecting sexual functioning—particularly loss of libido, vaginal dryness, and dyspareunia—were recurrent across several populations, including Indigenous Bolivian and Arab/Egyptian women, where urogenital discomfort and genital pruritus were common and directly linked to reduced sexual activity and avoidance of intercourse (22, 23). Among the Yoruba population in Nigeria, sexual withdrawal was even more pronounced: more than half of postmenopausal women reported discontinuing sexual activity, explaining this decision primarily through deeply rooted cultural beliefs that associate postmenopausal intercourse with illness, weakness, or spiritual harm (29).

In contrast, relational concerns were more prominent among Hispanic women, who reported that mood changes, reduced energy, and emotional exhaustion significantly interfered with intimacy, communication, and marital dynamics, beyond the impact of physical symptoms alone (21). These findings are consistent with multiethnic analyses in the United States, where Hispanic and African American women reported greater emotional distress, psychological symptoms, and diminished vitality—factors that indirectly affected partner relationships—compared with White, Chinese, or Japanese women (26, 31). The interplay between vasomotor symptoms, somatic discomfort, and emotional instability was also reflected in studies from India, where irritability, anxiety, poor concentration, and fatigue clustered with loss of sexual desire, suggesting that sexual and emotional domains during menopause tend to deteriorate concurrently rather than independently (25).

Experiences among Asian populations add further cultural nuance. Chinese and Singaporean Chinese women frequently described menstrual changes—especially irregular or heavy bleeding—as shame-inducing, disruptive, and anxiety-provoking events that affected body image, sexual confidence, and openness with partners (28, 32). For many, concerns about “abnormal” bleeding, fear of cancer, or the perception of premature aging promoted secrecy and sexual avoidance. Similarly, migrant Chinese women in Germany reported higher rates of sexual problems, exhaustion, and musculoskeletal discomfort compared with German women, suggesting that migration-related factors and cultural expectations intersect with menopausal changes to shape sexual experiences (24).

While menopause was interpreted as a loss—of youth, fertility, attractiveness, or desirability—in some cultures, other groups articulated more ambivalent or even positive narratives. Studies among Persian, Turkish, and North African women revealed mixed emotions regarding the cessation of fertility: some welcomed relief from reproductive responsibilities or the disappearance of menstrual discomfort, whereas others expressed concern that their husbands might regret the loss of reproductive capacity (33). Women from Lebanon, Morocco, Spain, and the United States similarly highlighted fatigue, physical strain, and diminished energy as major barriers to maintaining intimacy and everyday functioning, underscoring the universal—yet culturally filtered—nature of these midlife challenges (34).

In China, 17% of women explicitly reported a compromised sexual life and 13.8% noted relational difficulties with their husbands due to menopausal symptoms (28). Among Māori and non-Māori women in New Zealand, vaginal dryness and urogenital symptoms were frequently reported, although relational interpretations differed across ethnic groups (30). In nationally representative U.S. surveys, sexual disruption was more prevalent among African American and Caucasian women, who reported higher frequencies of palpitations, chest discomfort, and psychological distress—factors known to indirectly undermine intimacy (27).

### 3.7 Ethnic and cultural differences

Marked cross-ethnic variations were observed in both the perception and reporting of menopausal symptoms. In large multiethnic cohorts, such as those conducted in the United States, African American and Hispanic women consistently reported a higher symptom burden, including vasomotor and psychological symptoms, than White women (20, 31, 35). By contrast, Asian women (Chinese and Japanese) demonstrated the lowest prevalence of distress, frequently interpreting menopause as a natural and expected life transition rather than a medical condition (19, 26). Otras investigaciones asociaron diferencias conductuales y de autocuidado entre grupos étnicos (36).

Studies among Indigenous and Māori women highlighted culturally specific experiences that diverged from Western medical narratives. Māori participants from New Zealand often framed menopause as a continuation of the life cycle rather than pathology, though somatic discomforts such as leg cramps or urinary pain were frequent (30). Similarly, Indigenous Bolivian women associated menopause with “loss of blood” and diminished energy but also acknowledged it as a release from reproductive constraints (22).

Comparative studies revealed how migration and cultural adaptation shape menopausal experiences. Chinese migrant women living in Germany reported greater symptom severity than Chinese women in Beijing, suggesting the influence of acculturation and psychosocial stress (24). Likewise, in transnational analyses involving Lebanon, Morocco, Spain, and the United States, differences in fatigue and perceived health reflected both cultural norms and socioeconomic conditions (34).

### 3.8 Sociocultural determinants and meanings

The studies collectively illustrate that the meaning of menopause is culturally constructed, embedded in local belief systems, gender norms, and social expectations. In many African and Middle Eastern contexts, menopause was described as a stage of social withdrawal and loss of desirability, intertwined with religious and moral codes emphasizing modesty and purity (23, 29, 33). Conversely, in Indigenous and Asian cultures, cessation of menstruation was sometimes linked to wisdom, spiritual strength, and freedom from reproductive constraints, reflecting positive redefinitions of womanhood (22, 30).

Migration, modernization, and exposure to Western health models appeared to modify traditional interpretations. For instance, Chinese migrant women in Europe and Hispanic women in the United States increasingly adopted biomedical explanations for menopausal symptoms, associating them with stress, hormonal imbalance, or aging (21, 24). Meanwhile, North American women tended to frame menopause as a medicalized process requiring management, often seeking clinical validation for symptoms (20, 31).

On the other hand, the age at menopause onset demonstrates notable variation across ethnic groups. A comparative study reported a higher prevalence of early menopause among Korean women compared with women in the United States. Moreover, Korean women exhibited both a higher and consistently declining prevalence over time, indicating a lower risk of early menopause in younger cohorts relative to older generations within this population (45).

Social class, education, and marital status further influenced interpretations: women with lower socioeconomic status or limited access to healthcare were more likely to normalize discomfort and rely on cultural explanations, while those with higher education more frequently described menopause through a health literacy or empowerment discourse (25, 34), aunque la mayoría evidenció variaciones étnicas en la experiencia de la menopausia, algunos trabajos no reportaron diferencias significativas en actitudes según grupo étnico o nivel educativo (37). Detailed characteristics of each study, including country, population, design, analytical approach, and main outcomes, are presented in Table 3.

**Table 3:** Characteristics of included studies.

Author, year	Country, year and type of survey	Ethnic stratification, ethnic group and subgroup	Study design and participants	Analytical approach (AP)—Outcome (O)
Harlow et al. (20)	United States of America, 1995–1997.	Black and White American	Longitudinal, multi-racial/ethnic cohort study.	**AP:** Stratified Descriptive Analysis by ethnicity in disparities in the midlife health
	Local survey and semi structured interview in person		Size: 3,302 women	**O:** Black women report earlier menopause, more vasomotor symptoms, and greater chronic disease burden compared to White women.
			Age: 40–55 years.	
Okonofua et al. (29)	Nigeria, 1989	Yoruba	Cross-sectional study.	**AP:** Stratified Descriptive Analysis by ethnicity in age of menopause and incidence of menopausal symptoms
	Local survey		Size: Women	**O:** When analyzing attitudes toward menopause, 94% of respondents indicated they still felt adequate as women, while 6% said they felt inadequate. A more detailed analysis showed that of those who felt inadequate, 6% were childless. Furthermore, 58% of women admitted to having stopped having sex after reaching menopause. This was because most believed that sexual intercourse after menopause could cause illness.
			Age: 39–62 years	
Avis et al. (31)	United States of America, 1996–1997.	White, Black, Hispanic, Chinese, Japanese, other	Cross-sectional study.	**AP:** Stratified Descriptive Analysis by ethnicity in Health-related quality of life.
	Telephonic survey and semi structured interview		Size: 3,302 women	**O:** Ethnic group was significantly related to impaired functioning on all scales. Except for the Role–Emotional domain, Hispanic women were most likely to report impaired function. Chinese and Japanese women were least likely to report impaired function for all domains. Compared with whites, blacks were more likely to report impaired functioning on the Pain and Social Functioning domains and less likely than whites to report impairment on Vitality.
			Age: 42–52 years	
Avis et al. (19)	United States of America, 1995–1997.	Caucasian, African-American, Chinese, Japanese, and Hispanic	Cross-sectional study.	**AP:** Stratified Descriptive Analysis by ethnicity in menopausal status and symptoms
	Telephonic survey and semi structured interview		Size: 16,065 women	**O:** Racial/ethnic differences in symptom reporting, as well as differences by menopausal status. Caucasian women reported more psychosomatic symptoms. African-American women reported more vasomotor symptoms.
			Age: 40–55 years	
Chou et al. (28)	China, 2011–2012	Asian	Cross-sectional study	**AP:** Stratified Descriptive Analysis
	Standardized survey		Size: 442 women	**O:** Menopausal symptoms affected QoL in 57.2% of women: daily life in 36.7%, work in 29.2%, sexual life in 17.0%, and relationship with husband in 13.8%. Daily life was significantly affected by hot flushes and joint/muscular discomfort; work was reportedly affected by irritability and exhaustion; sexual life was reported to be affected by hot flushes, sexual problems, and vaginal dryness, and relationship with husband was affected by sexual problems
			Age: 40–60 years	
Hinrichsen et al. (24)	Germany, 2005–2008	Native German women in Berlin, migrant Chinese women in several German cities, Chinese women in Beijing	Cross-sectional study	**AP:** Descriptive Reporting, Hypothesis Testing for Frequencies, Factor Analysis for Latent Symptoms, and Logistic Regression for Risk Factor Influences
	Standardized survey		Size: 1,000 German women and 852 Asian	**O:** The results of the study reveal several significant differences between the groups, particularly with regard to the stated severity of menopausal symptoms, with sociocultural aspects—particularly migration experience—possibly playing a role here. For the migrant Chinese women in Germany these were exhaustion, sexual problems and joint/ muscle discomfort, and the Chinese women in Beijing suffered most frequently from heart discomfort, sleep problems and hot flushes.
			Age: 45–60 years	
Schnatz et al. (21)	United States of America, 2002–2004	Hispanic and	Prospective study	**AP:** The data were analyzed for frequencies of menopausal symptoms, the most common symptoms reported, women's perceptions of which symptoms affected them the most, and their level of understanding about menopause.
	Local survey			**O:** When comparing Hispanic women with Caucasian women, statistically significant differences (*P* < 0.05) were observed in symptoms, including mood changes (76% H, 54% C), a decrease in energy (56% H, 36% C), palpitations (54% H, 26% C), breast tenderness (39% H, 28% C), memory loss (34% H, 22% C), and vaginal dryness (34% H, 44% C). After controlling for education and income, differences persisted in mood changes, decreased energy, and palpitations between the groups. The symptoms reported by postmenopausal Caucasian women in this sample align with rates from the literature, whereas postmenopausal Hispanic women reported several symptoms at higher frequencies. These differences remained evident even after accounting for socioeconomic factors.
		Caucasian women	Size: 404 women	
			Age: 40–90 years	
Castelo-Branco et al. (22)	Bolivia, 2002	Indigenous of South America	Cross-sectional study	**AP:** Stratified Descriptive Analysis
	Local survey and structured interviews in person		Size: 125 women	**O:** Loss of libido was the primary menopausal complaint, reported by 51% of the interviewed women, followed closely by hot flushes (45%), genital itching (40.8%), and dyspareunia (40%), which were also frequently endorsed. In a regression analysis examining vital events, premenstrual symptoms, and vasomotor symptoms as independent variables, with anxiety, depression, somatic symptoms, and loss of libido as dependent variables, vasomotor symptoms emerged as the strongest predictor of risk for psychological and somatic climacteric symptoms. Consequently, these women exhibited an elevated risk for anxiety, depression, somatic symptoms, and loss of libido, underscoring the central role of vasomotor disturbances in broader menopausal symptomatology.
			Age: 35–54 years	
Dasgupta et al. (25)	India, 2011–2012	Hindu ethnic group of West Bengal	Cross-sectional study	**AP**: Stratified Descriptive Analysis
	Standardized survey		Size: 1,400 women	**O:** The analysis of menopausal symptoms highlighted strong interconnections, particularly around mood-related perceptions, where issues like anxiety, depression, irritability, and tiredness formed a core psychological-somatic cluster. Tiredness, for instance, was closely linked to irritability, anxiety, joint pain, and numbness of extremities, while poor concentration and sleep disturbances further amplified mood disruptions, often co-occurring with loss of sexual desire and headaches. This grouping underscores how vasomotor and somatic complaints, such as hot flashes and rapid heartbeat, exacerbate emotional instability, suggesting that mood changes in menopause are not isolated but intertwined with broader symptom networks, warranting integrated therapeutic approaches to restore emotional balance.
			Age: 40–55 years	
Bromberger et al. (26)	United States of America, 1995–1997	African American, White, Chinese, Hispanic, and Japanese	Cross-sectional study	**AP:** Stratified Descriptive Analysis
	National representative Survey and Semi-Structured interviews by telephone or in person		Size: 16,065 women	**O:** White, Hispanic, and African American women reported the highest rates of distress, followed by Chinese and Japanese women. Subgroup analyses indicated differences in psychological distress rates, though not statistically significant, between Japanese women educated in the United States and those educated elsewhere.
			Age: 40–55 years	
Im et al. (27)	United States of America, **2006–2011**	**African American, Caucasian, Hispanic, Chinese, and Japanese** women	Cross-sectional study	**AP:** Multiple logistic regression and ANOVA to examine racial/ethnic differences in cardiovascular symptoms controlling for covariates (age, BMI, socioeconomic factors, menopausal status).
	National representative survey.		Size: 542 women	**O:** Prevalence and severity of cardiovascular symptoms (palpitations, chest pain, shortness of breath). African American and Caucasian women reported higher prevalence; Asian women reported lower frequency; Hispanic women showed stronger association with stress.
			Age: 40–60 years.	
Abou-Raya et al. (23)	Egypt, 2013–2014	**Arabs from urban and rural areas**	Cross-sectional study	**AP:** Stratified Descriptive Analysis
	Standardized survey and structured interviews in person		Size: 540 women	**O:** Most frequently reported symptoms were as follows: joint and muscular discomfort (501, 92.8%), urogenital symptoms (460, 85.2%), physical and mental exhaustion (416, 77.0%), hot flushes (371, 68.7%), irritability (368, 68.1%), depressive mood (360, 66.7%), anxiety (339, 62.8%), and sleeping problems (315, 58.3%).
			Age: 40–65 years	
Ylitalo et al. (35)	United States, 1996 and 2010	Caucasian, African American, Chinese, Japanese	Longitudinal study	**AP:** Longitudinal analysis
	National representative survey.		Size: 2,497 women	**O:** African American women were more likely to report substantial limitations (OR = 1.63; 95% CI:1.06, 2.52) and Chinese women were more likely to report some limitations (OR = 2.03; 95% CI: 1.22, 3.36) compared to Caucasian women.
			Age: 42–52 years	
Wang et al. (36)	United States of America, 1988–1994	Non-Hispanic Black,	Cross-sectional study	**AP:** Cross-sectional multivariate analysis with linear regressions stratified by ethnicity
	National representative survey.	Non-Hispanic White, Mexican-American	**Size**: 2,905 women	**O:** Positive associations with estrogen use, calcium intake and physical activity, and a negative association with smoking, were noted in White women.
			Age: ≥60 years	
Wilbur et al. (37)	United States of America, 1995	African American, White, Hispanic, Asians	Cross-sectional study	**AP:** Stratified Descriptive Analysis
	Standardized survey		Size: 149 women	**O:** No differences in menopausal attitude were demonstrated based on ethnicity, occupation, and education.
			Age: 35–65 years	
Lawton et al. (30)	New Zealand, 1999–2004	Maori indigenous and non maori	Cross-sectional study	**AP:** Cross-sectional analysis of data collected during recruitment of women from 27 primary-care practices into an observational study and the international WISDOM trial of postmenopausal.
	Standardized survey and structured interviews in person		Size: 3,616 women	**O:** Non-Maori women more frequently reported insomnia and vaginal or genital dryness in the past 4 weeks (*p* 50.05). Maori women more frequently reported cramping in one leg, swelling in one or both legs, pain or burning during urination, and abdominal cramps (*p* 50.05). Overall, 36.5% of women experienced vasomotor symptoms (1,319/ 3,616), 88.3% of women experienced psychological symptoms (3,194/3,616), and 45.2% experienced vaginal symptoms (1,319/3,616) in the past 4 weeks.
			Age: 49–70 years	
Lim et al. (32)	Singapore, 2010	Chinese Singaporean	Cross-sectional study	**AP:** Thematic analysis was used to analyze interviews.
	Structured interviews in person		Size: 14 women	**O:** The most prevalent symptom reported by participants during the menopausal transition was abnormal bleeding, which manifested in two primary forms: prolonged heavy bleeding that raised concerns about anemia and necessitated interventions like dilation and curettage, as one woman described her nonstop, pad-changing-every-two-hours episodes that recurred despite treatment; and irregular periods with shortened intervals or unpredictable flow, duration, and timing, leading to feelings of messiness and disruption. Many women expressed anxiety over potential pathological causes, such as cancer, prompting swift medical consultations, especially among those in their mid-40s who viewed the changes as premature for menopause.
			Age: 40–60 years	
Maoz et al. (33)	Israel, 1977	Central European, Persian, Turkish, North African, Arab	Cross-sectional study	**AP:** Cross-sectional study with interviews and medical examination.
	Structured interviews in person		Size: 1,148 women	**O:** Regarding fertility, a clear majority in each group welcomed its cessation, especially Persians, Turks, and Europeans. However, the only statistically significant difference overall was that Arabs and North African Jews were more concerned, in relative terms, with their husbands' regret over having lost the ability to have more children. These two groups, and North Africans in particular, tend to express greater regret over the cessation of fertility in all other respects as well. The Arabs, much more than others, felt that there was now the possibility of improving their physical health. However, traditional Jewish groups were concerned about the possibility of “weakness and illness,” as well as a sense of aging, a concern less characteristic of Europeans.
			Age: 45–54 years	
Choe et al. (45)	United States of America, 1999–2014 and Korea, 2007–2012	US Hispanic, US Non-Hispanic white, US Non-Hispanic black, Korean	Retrospective study	**AP:** The prevalence of early and precocious menopause was analyzed in three ethnic groups in the United States and Korea.
	National representative survey.		Size: 9,209 US women and 9,828 Korean women	**O:** US non-Hispanic white women had the lowest prevalence of premature (1.7%) and early (3.2%) menopause, and Koreans had the highest (2.8% of premature and 7.2% of early menopause).
			Age: ≥45 years	
Parsons et al. (34)	Lebanon, Morocco, Spain, and the United States of America, 1997–2002	People from Lebanon, Morocco, Spain, and the United States of America	Comparative study	**AP:** Statistical analyses were used to assess the determinants of reporting poor health, and textual analyses were used to highlight themes related to perceptions of health.
	Structured interviews in person		Size: 1,190 women	**O:** Women from diverse countries shared poignant accounts of midlife health challenges, predominantly centered on fatigue, diminished energy, and physical limitations. A Lebanese participant described her health as generally good but burdened by intense pain when tired or angry, noting her inability to complete household tasks in one go and lamenting how age had eroded her former strength. In Morocco, another woman expressed feeling persistently ill and weak, underscoring a broader sense of frailty. Spanish testimonies highlighted exhaustion from juggling work, childcare, and meal preparation, leaving her utterly drained by day's end. Similarly, an American woman reflected on her transformation, feeling markedly more tired and less energetic than before.
			Age: 45–55 ages	

## 4 Discussion

This review highlights the limited body of scientific research addressing menopausal experiences among Indigenous peoples in Latin America and Africa, revealing a structural gap in cross-cultural studies on women's health. Most available research focuses on urban populations from industrialized countries—often involving Caucasian or Asian migrant women—while the voices of Indigenous, Afro-descendant, and rural women remain largely underrepresented (22, 23, 29). Such asymmetry restricts the understanding of menopause as a socially situated phenomenon, obscuring the spiritual, communal, and alternative cosmological frameworks through which many women interpret this life stage.

The reviewed studies consistently showed that the perception, interpretation, and management of symptoms do not constitute a homogeneous biological process, but rather a transition shaped by the intersection of cultural, ethnic, and social factors. These determinants influence both the expression of symptoms and their symbolic and moral meanings (20, 25, 30). Recent qualitative syntheses reinforce this view: meta-ethnographic evidence indicates that sexual, emotional, and relational experiences during menopause are deeply mediated by sociocultural scripts around femininity, aging, and marital expectations (38). Similarly, a comprehensive synthesis of qualitative research identified that cultural beliefs profoundly shape how women negotiate changes in sexual desire, orgasm, and intimacy, and how they interpret the meaning of menopause within their communities (39).

Ethnic differences in menopausal experiences underscore the profound influence of sociocultural determinants on physical, psychological, and sexual symptoms. In the United States, African American and Latina women experience a higher burden of vasomotor, psychosomatic, mood-related, and fatigue-related symptoms, whereas Asian women—particularly Chinese and Japanese—report lower symptom intensity and a more naturalized perception of the transition, mediated by philosophical conceptions of bodily balance (20, 26, 27, 31). Studies from Taiwan, China, Australia, Iran, and Sweden show that menstruation is often perceived as a marker of femininity, and its cessation may be interpreted as a loss of womanhood, leading to reduced sexual interest, altered body image, and feelings of incompleteness; in the same synthesis, women from Australia expressed ambivalent views, with some valuing freedom from menstruation and pregnancy concerns, while others experienced menopause as an unwelcome sign of aging (39).

In Latin America, Indigenous and Hispanic migrant women describe menopause as an interdependent bodily and emotional experience, intertwined with the loss of reproductive roles, the symbolic weight of female sexuality, and socioeconomic vulnerability (21, 22). Qualitative findings across the Middle East and North Africa reveal that women commonly attribute menopausal changes—especially fatigue, anxiety, and urogenital symptoms—to moral and religious frameworks that reinforce sexual restraint, modesty, or abstinence (23, 29, 33). A recent Saudi Arabian study further demonstrates that women's intimate lives are shaped not only by biological changes but also by culturally embedded expectations concerning modesty, marital duty, and perceived attractiveness, emphasizing how aging affects body image, self-confidence, and couple dynamics (38).

Evidence from Asia and Oceania highlights narratives of acceptance in cultures where menopause is incorporated into a natural or spiritual life cycle (24, 32). Māori and non-Māori women, for example, acknowledge significant urogenital symptoms but interpret them within broader cultural frameworks of aging, resilience, and communal support (30). Taken together, the evidence suggests that women belonging to historically marginalized ethnic groups experience a greater psychosomatic and emotional burden, modulated by structural inequalities, gender norms, and cultural representations of aging. In contrast, cultures grounded in holistic or communal worldviews tend to integrate menopause as a vital transition that is not pathologized.

Scientific evidence demonstrates that women belonging to historically marginalized ethnic groups experience a greater psychosomatic and emotional burden during the menopausal transition, influenced by structural and sociocultural factors. In the Study of Women's Health Across the Nation (SWAN), African American women exhibited more severe and prolonged vasomotor and mood symptoms than White women, findings linked to socioeconomic inequalities and structural racism (20). Similarly, higher frequencies of cardiovascular symptoms have been documented among ethnic minorities—alongside lower clinical recognition of these symptoms (27),—as well as disparities in physical performance mediated by social factors such as education and financial strain (40). Globally, a recent meta-analysis identified a significantly higher prevalence of depressive and urogenital symptoms in South America, reinforcing the role of social and cultural determinants in the symptomatic expression of the climacteric (41). Collectively, these findings indicate that the menopausal experience is deeply shaped by structural inequities, gender norms, and ethnocultural contexts.

Within the broader framework of health inequities, only six studies explicitly identified gaps in access to healthcare, use of hormonal therapies, or availability of resources for symptom management—reflecting a persistent structural bias within health systems. In the United States, research by Harlow et al. (20), Avis et al. (31), Bromberger et al. (26), and Im et al. (27) shows that non-Hispanic White women have greater access to specialized medical services and hormone replacement therapy, whereas African American and Hispanic women report lower access, greater reliance on home remedies, or complete absence of formal treatment for symptom control (20, 26, 27, 31). In the same context, Schnatz et al. (21) found that even after adjusting for education and income, Hispanic women continued to experience higher symptom burden and lower medical guidance—suggesting the coexistence of cultural barriers and institutional discrimination (21).

Similarly, the study by Wang et al. (36) revealed that White American women were more likely to receive estrogen therapy and calcium supplementation, while African American and Mexican American women showed lower prescription and clinical follow-up rates. In contrast, research conducted in non-Western contexts—such as Abou-Raya et al. (23) in Egypt and Hinrichsen et al. (24) among Chinese migrants in Germany—revealed fragmented and culturally insensitive care, where ethnic minority and migrant women faced limited access to gynecological services and insufficient medical information in their language or aligned with their beliefs (23, 24). Findings from integrative and qualitative global reviews underscore that inadequate knowledge, low health literacy, and insufficient clinical guidance are widespread, especially in low-resource settings (42, 43).

Taken together, these findings reveal that privileges in access to menopausal care and treatment are concentrated among urban, White, and higher socioeconomic status women, while racial, Indigenous, and migrant minorities face structural inequalities that deepen health inequities during the climacteric.

In light of the persistent inequalities in access to healthcare and treatment during menopause, it is essential to move beyond reductionist biomedical approaches and to integrate holistic and community-based worldviews that conceive this stage as a non-pathologized vital transition. Evidence indicates that women living in contexts where menopause is interpreted within symbolic and collective frameworks experience lower symptom severity and reduced dependence on hormonal treatments, fostering a more balanced and adaptive coping process (24, 30).

According to Lock and Kaufert (44), the medicalization of menopause is a Western cultural construct rather than a universal response to reproductive aging. Health professionals must recognize that the perception, interpretation, and management of menopausal symptoms do not constitute a homogeneous biological process, but rather an experience mediated by the intersection of cultural, ethnic, and social factors. Incorporating these perspectives into clinical practice and health policies involves not only improving equitable access to healthcare resources but also promoting intercultural care and respect for local knowledge systems—thus ensuring a more comprehensive and humanized understanding of menopause.

### 4.1 Outlook

Menopause is not only a time of loss but also a period of growth, self-realization, and transformation for women. Cultural analysis of menopause indicates that women's empowerment depends on their ability to make decisions within the context of inequalities, cultural barriers, and limited access to healthcare services. In the populations studied, the greatest psychosomatic and emotional burden is associated with persistent inequities and culturally insensitive healthcare services, which in many cases restrict women's autonomy in managing their symptoms and health appropriately.

However, in cultures where menopause is viewed as a natural stage of life, strategies adapted to their environment are adopted. This approach fosters a space to redefine women's roles and seize the opportunity to change traditional expectations, incorporating cultural practices and improving the understanding of health information. Moving toward empowerment requires reducing inequalities, providing culturally appropriate care, and creating spaces for participation. From this perspective, menopause can be reconfigured as a stage that allows for autonomy, physical well-being and growth, and not just as a period of loss.

### 4.2 Recommendations

Future research on menopausal experiences across ethnic groups should expand in several strategic directions to address the structural gaps identified in this review. First, there is a critical need for studies focusing on Indigenous, Afro-descendant, and rural women from Latin America, Africa, and other underrepresented regions. The limited evidence available for these groups restricts the understanding of how cosmological beliefs, collective practices, and intergenerational knowledge shape the menopausal transition. Future investigations should therefore prioritize culturally grounded methodologies and community-engaged approaches that capture local explanatory models and epistemologies surrounding menopause.

Research designs must move beyond the dominance of cross-sectional quantitative surveys to include longitudinal, mixed-methods, and qualitative frameworks capable of tracing symptom trajectories, cultural transformations, and temporal changes in women's perceptions. Longitudinal designs would help clarify how structural determinants—such as socioeconomic mobility, migration, acculturation, gender norms, and exposure to biomedical models—influence symptom expression over time. Mixed-methods studies may also enhance interpretive depth by integrating experiential narratives with clinical or epidemiological indicators.

There is a need to systematically explore the role of structural inequities—such as racism, discrimination in healthcare, economic precarity, and limited access to gynecological services—in shaping symptom burden and health-seeking behavior among minority ethnic groups. Future studies should incorporate intersectional analyses capable of capturing how ethnicity interacts with social class, education, sexuality, disability, and geographic isolation to produce differentiated menopausal trajectories. Future reviews and primary studies should incorporate information retrieved through manual searches or community-generated evidence, provided that methodological rigor is preserved. This complementary inclusion would allow a more comprehensive understanding of underrepresented cultural contexts that are seldom present in mainstream academic databases.

Culturally adapted clinical tools and intercultural models of menopausal care should be developed and validated specifically for minority and rural populations. Research evaluating the acceptability, feasibility, and effectiveness of these models—particularly those integrating community health workers, traditional knowledge systems, or non-pharmacological practices—would support the creation of more equitable, context-sensitive health interventions.

### 4.3 Strengths and limitations

Differences in the intensity and meaning of menopausal symptoms across ethnic groups reflect the intersection of cultural, religious, socioeconomic, and gender-related factors. African American and Hispanic women experience greater vasomotor and psychological symptom burdens, whereas Asian and Indigenous women tend to reframe menopause as a natural transition, illustrating the influence of cultural context on bodily perception and distress.

This review reveals a marked lack of research on menopause among Indigenous women in Latin America and Africa, limiting the understanding of the phenomenon from an intercultural perspective. This absence reproduces inequalities in scientific production and perpetuates a hegemonic approach centered on urban White women from industrialized countries, thereby obscuring the cultural and community-based determinants that shape menopausal experience.

It should also be noted that during the literature search, a few potentially relevant studies were identified through manual exploration but were not incorporated into the final synthesis to preserve the methodological rigor and reproducibility of the systematic process. Although this decision ensured adherence to the Joanna Briggs Institute's scoping review framework, it may have excluded additional evidence of contextual value. Future reviews are encouraged to include such manually retrieved studies through a complementary or sensitivity analysis, to capture all relevant perspectives and enhance the comprehensiveness of the evidence base.

## 5 Conclusions

The findings confirm that women belonging to racial, Indigenous, or migrant minorities face systemic barriers in accessing medical care and hormonal therapies, in contrast to the privileges enjoyed by urban, White, and higher socioeconomic status women. These gaps underscore the need for health policies that are sensitive to cultural diversity and to the social determinants of health.

Overcoming the biomedical paradigm requires recognizing that menopause is not a uniform biological process but a transition shaped by diverse local worldviews and historical experiences. Integrating holistic and community-based approaches into healthcare practice will enable the development of more equitable, humanized, and culturally respectful care models—acknowledging menopause as a natural stage in the life cycle rather than as a disease.

## Data Availability

The original contributions presented in the study are included in the article/Supplementary Material, further inquiries can be directed to the corresponding author.
